# Evaluation of muscle strength and motor abilities in children with type II and III spinal muscle atrophy treated with valproic acid

**DOI:** 10.1186/1471-2377-11-36

**Published:** 2011-03-24

**Authors:** Illora A Darbar, Paulo G Plaggert, Maria Bernadete D Resende, Edmar Zanoteli, Umbertina C Reed

**Affiliations:** 1 Department of Neurology, Medical School of the University of São Paulo, São Paulo, Brazil; 2 Neuromuscular Section, Associação de Assistência à Criança Deficiente, São Paulo, Brazil

## Abstract

**Background:**

Spinal muscular atrophy (SMA) is an autosomal recessive disorder that affects the motoneurons of the spinal anterior horn, resulting in hypotonia and muscle weakness. The disease is caused by deletion or mutation in the telomeric copy of *SMN *gene (*SMN1*) and clinical severity is in part determined by the copy number of the centromeric copy of the *SMN *gene (*SMN2*). The *SMN2 *mRNA lacks exon 7, resulting in a production of lower amounts of the full-length SMN protein. Knowledge of the molecular mechanism of diseases has led to the discovery of drugs capable of increasing SMN protein level through activation of *SMN2 *gene. One of these drugs is the valproic acid (VPA), a histone deacetylase inhibitor.

**Methods:**

Twenty-two patients with type II and III SMA, aged between 2 and 18 years, were treated with VPA and were evaluated five times during a one-year period using the Manual Muscle Test (Medical Research Council scale-MRC), the Hammersmith Functional Motor Scale (HFMS), and the Barthel Index.

**Results:**

After 12 months of therapy, the patients did not gain muscle strength. The group of children with SMA type II presented a significant gain in HFMS scores during the treatment. This improvement was not observed in the group of type III patients. The analysis of the HFMS scores during the treatment period in the groups of patients younger and older than 6 years of age did not show any significant result. There was an improvement of the daily activities at the end of the VPA treatment period.

**Conclusion:**

Treatment of SMA patients with VPA may be a potential alternative to alleviate the progression of the disease.

**Trial Registration:**

ClinicalTrials.gov: NCT01033331

## Background

Spinal muscular atrophy (SMA) is an autosomal recessive neurodegenerative childhood disease characterized by loss of lower motor neurons in the anterior horn cells in the spinal cord and brainstem, causing progressive proximal symmetrical weakness and atrophy of skeletal muscle. It is the most common inherited neuromuscular diseases of childhood with a prevalence of 1 in 6,000 to 10,000 [1,2]. SMA has been classified clinically into four types based on age of onset and maximum function attained [3]. Type I SMA, Werdnig-Hoffmann disease, is characterized by onset within the first 6 months of age, and the children never achieve the ability to sit without support. Type II SMA is characterized by onset of muscle weakness and hypotony usually after six months of age; the children are able to sit but unable to walk unaided. In the type III SMA, Kugelberg-Welander disease, the initial manifestations usually occurs after 18 months of age; and the patients achieves the ability to walk. Type IV SMA is a mild form with an adult onset of muscle weakness. All four types of SMA show a progressive course, which is severe in type I and mild in type III and IV SMA [4].

Approximately 95%-98% of individuals with a clinical diagnosis of SMA lack exon 7 in both copies of *SMN1 *gene, while approximately 2%-5% of them are compound heterozygotes for deletion of *SMN1 *in one allele and an intragenic mutation of *SMN1 *in another allele [5,6]. The human *SMN *gene is located at the chromosome 5 (5q), and exists in two copies, *SMN1 *and *SMN2*. *SMN1 *is the telomeric copy and produces a full-length survival motor neuron (SMN) protein necessary for a normal lower motor neuron function [5]. The centromeric *SMN2 *copy mostly encodes a protein that is lacking in exon 7 due to alternative splicing, thereby producing a less stable protein. The amount of functional SMN protein produced by *SMN2 *is not sufficient to prevent the progressive degeneration of motor neuron when *SMN1 *is absent. The *SMN2 *gene copy number is variable, ranging from zero to five. Several studies have demonstrated a strong inverse correlation between the number of *SMN2 *copies and SMA severity [7-11]. The presence of three or more copies of *SMN2 *is correlated with a milder phenotype [9-11]. Thus, *SMN2 *activation and/or modulation of the *SMN2 *splicing pattern have been used as a strategy for SMA treatment to increase full length SMN protein level.

The modulation of *SMN2 *expression is in part controlled by acetylation and deacetylation of histones in the promoter region. Thus, the inhibitors of histone deacetylases (HDAC), such as phenylbutyrate and valproic acid (VPA), could promote the acetylation of the DNA increasing the gene expression and the level of full-length SMN protein [12]. Many studies have demonstrated that phenylbutyrate, a drug used in the treatment of urea acid cycle disorders, and VPA increase *SMN2 *transcripts and full-length SMN protein in different types of cells from patients and in animal models of SMA [13-16]. Preliminary clinical trials have studied the preclinical stages of research in animal models or in vitro experiments [17-22]. Brichta et al [17] observed an increase of 13 times in the blood level of full length SMN proteins in seven SMA children treated with VPA. An improvement in seven adult patients with SMA type III and IV during treatment with VPA was reported by Weihl et al [18]. Tsai et al [19] observed significant increase of the overall strength in type II SMA treated with VPA during six months. In an open label study, Swoboda et al [20] assessed 42 SMA individuals treated with VPA during 12 months, and observed a significant improvement almost restricted to participants aged less than 5 years. More recently, Swoboda et al [21] assessed 61 subjects randomized 1:1 to placebo or treatment with VPA and L-carnitine for six months, but no benefit was demonstrated. Thus, the clinical efficacy of VPA in patients with SMA is still controversial. In this study, we quantified the muscular strength and motor function of patients with SMA type II and III treated with VPA and assessed the side effects of the medication.

## Methods

### Study design

We included in this study 35 patients with SMA type II and III confirmed by molecular analysis, aged between 2 to 18 years old, regularly treated at the Out-patient Service Center for Neuromuscular Disorders and Child Neurology at our Institution. This open, comparative and longitudinal study had the approval of the Ethical Committee for Project and Research; and the parents or legal guardians signed an informed consent form for participating in the study. The patients were evaluated five times during one year by the same examiners: PGP (physician) and IAD (physical therapist). The first evaluation was done just before the prescription of VPA, and the following assessments were carried out at three, six, nine and twelve months after the onset of VPA therapy. All patients received 20 mg/Kg/day of VPA (divided into 2 or 3 doses), L-Carnitine (100 mg/Kg/day) and vitamin supplement. Laboratory tests were performed before the beginning of the treatment and after 6 and 12 months, and included the measurement of blood cells, hepatic and pancreatic enzymes and serum level of VPA.

### Muscular strength and functional assessment

Patients were evaluated using the following methods: 1) The Hammersmith Functional Motor Scale (HFMS), which includes 20 items scored from 0 to 40 [23]; 2) the Medical Research Council (MRC) method for testing the muscular strength of the main muscular groups (flexion and extension of shoulders, hips, elbows, wrists, knees, feet and trunk; abduction and adduction of shoulders and hip), scored from 0 to 5 points [24]; and 3) the Barthel Index for evaluating the daily activities in patients older than 5 years of age [25].

#### Statistical analysis

We used the ANOVA test to compare both the averages by time and groups. The Pearson product-moment correlation coefficient was calculated with its interval at 95% (IC 95%) to analyze the relation between the interested variables.

## Results

Among the thirty-five patients with SMA, five did not return regularly to the appointments and eight did not take the drug according to the medical prescription. Thus, only 22 patients, aged between 2 and 18 years (mean = 5.5), 14 SMA type II (63.6%) and 8 type III (36.4%), completed the period of the study (Table 1). In all 22 patients the serum concentration of VPA was between 40-100 mg/dL in the period of the study. No patient displayed any significant change in laboratory tests performed during the period of follow-up. Five patients developed a worsening of prior hand tremors, and three patients had their body mass index (BMI) increased during the treatment, but not reaching the overweight category.

**Table 1:** General data from 22 patients with SMA treated with VPA.

Case	Age/sex	SMA type	Age at diagnosis	Maximal motor ability	Procedures
1	9y/F	II	8y	Sits	------------
2	3y/F	II	1y	Sits	------------
3	5y/M	III	4y	Walks independently	------------
4	6y/M	III	2y	Walks independently	------------
5	18y/F	III	4y	Walks independently	------------
6	3y/F	II	1y	Sits	------------
7	13y/M	III	4y	Walks independently	------------
8	2y/F	III	2y	Walks independently	------------
9	6y/M	II	3y	Stand up with support	Foot and spinal orthesis
10	5y/M	II	10 mo	Sits	-----------
11	15y/F	II	2y	Sits	Back surgery
12	5y/M	III	4y	Walks independently	-----------
13	13y/F	II	3y	Sits	Back surgery
14	3y/F	III	8 mo	Walks independently	-----------
15	3y/M	II	1y	Sits with support	-----------
16	8y/M	III	1y	Walks independently	-----------
17	15y/F	II	2y	Sits	Back and hip surgery
18	3y/F	II	8 mo	Sits	Spinal orthesis
19	11y/M	II	1y	Sits	Foot orthesis
20	2y/M	II	2y	Sits with support	Foot orthesis/BiPAP
21	4y/M	III	1y	Walks independently	Foot orthesis
22	16y/M	II	7y	Sits with support	--------------------

F = female, m = male, y = year, mo = months

### Muscle strength, the HFMS and the Barthel index

The comparison between the MRC mean in the first evaluation and in subsequent ones did not show any significant difference (p > 0.05) (Table 2). The analysis of all 22 patients together showed a significant difference in the HFMS scores comparing the first with the second and the fourth evaluations (p = 0.038 and p = 0.039, respectively), but not in the third and fifth evaluations. The group of children with SMA type II presented a significant gain in HFMS score comparing the first evaluation with the subsequent ones (p = 0.031) (Table 3). This gain in HFMS score was not observed in the group of type III patients (p = 0.961). Patients younger than six years of age had a better mean score on HFMS than the group older than six years of age (Table 4). However, the comparison of the HFMS score of the first evaluation with the subsequent ones in the groups of patients younger and older than 6 years of age did not show any significant result (p = 0.240 and p = 0.922, respectively) (Table 4). According to the Table 2, there was a significant difference when comparing the mean of the Barthel score of the first evaluation with the fourth and fifth evaluations, indicating an improvement of the daily activities at the end of the VPA treatment period.

**Table 2:** Comparison between the mean of the MRC, HFMS and Barthel index scores in the first evaluation and the subsequent evaluations in the 22 SMA patients treated with VPA.

Evaluation	MRC	HFMS	Barthel
1 × 2	-0.0 ± 0.4 (p = 0.999)	-0.8 ± 0.4 (p = 0.038)	-0.0 ± 0.7 (p = 0.999)
1 × 3	-0.1 ± 0.6 (p = 0.947)	-1.0 ± 0.5 (p = 0.056)	-1.3 ± 0.9 (p = 1.53)
1 × 4	-0.4 ± 0.7 (p = 0.600)	-1.4 ± 0.6 (p = 0.039)	-2.3 ± 1.1 (p = 0.043)
1 × 5	-1.0 ± 0.8 (p = 0.267)	-1.4 ± 0.7 (0.063)	-2.7 ± 1.3 (p = 0.045)

**Table 3:** Evolution of the HFMS scores in the two SMA groups (type II and III) during the VPA treatment.

	Evaluations					
		
SMA	1	2	3	4	5	p (*)
Type II	13.6 ± 10.0	14.9 ± 11.4	15.5 ± 11.1	16.0 ± 11.2	16.9 ± 10.6	0.031
Type III	24.9 ± 3.4	24.8 ± 3.3	24.4 ± 3.5	24.4 ± 3.5	24.1 ± 3.9	0.961
p (**)	0.006	0.016	0.029	0.039	0.051	

* Comparison between the first evaluation with the subsequent ones

** Comparison between both groups (type II and III) in each evaluation

**Table 4:** Evolution of the HFMS scores in the two age groups of SMA patients during the VPA treatment.

	Evaluations					
		
Age	1	2	3	4	5	p (*)
≤ 6 years	21.5 ± 8.8	22.5 ± 9.5	22.9 ± 8.7	23.2 ± 8.3	23.5 ± 8.7	0.240
> 6 years	12.2 ± 8.8	12.7 ± 9.0	12.8 ± 9.0	13.0 ± 9.2	12.8 ± 9.1	0.922
p (**)	0.018	0.012	0.010	0.009	0.007	

* Comparison between the first evaluation with the subsequent ones

** Comparison between both groups of patients in each evaluation

## Discussion

Our present study demonstrated that there was not any evident motor function deterioration during the period of one year of treatment with VPA in patients with SMA type II and III. We did not detect any significant change in muscular strength tested using the MRC scale. However, we observed a significant improvement in the motor abilities assessed by HFMS, a scale that was structured and validated specifically for evaluating children with SMA [23]. When all 22 patients were analyzed together, the comparison of the first HFMS score with the following ones showed a significant improvement only in the second and fourth evaluations, but not in the third and fifth ones, suggesting that treatment with VPA might produce variable pick of action. Another way, when both groups of SMA (type II and type III) were analyzed separately, there was a significant gain on HFMS score comparing the first evaluation with the subsequent ones only in the SMA type II group.

The major limitation of our study was the non inclusion of a control group. Despite suffering from a progressive neurodegenerative disease, patients with SMA type II/III may show short term improvement of their motor status, in which it could be influenced by many factors including physical therapy, mental status and systemic diseases. However, in the period of evaluation, we could observe that no patient presented deterioration of their motor function, in which it would be expected considering the progressive course of the disease.

In our study, we did not perform *SMN2 *mRNA level analysis. However, studies assessing *SMN *mRNA levels in treated patients with VPA have demonstrated conflicting results. Brichta et al [17] observed elevated *SMN2 *mRNA levels in seven patients with SMA treated with VPA and unchanged or decreased levels in thirteen patients. Swoboda et al [20] did not show any significant increase of *SMN*2 mRNA levels in children treated with VPA, although the level of mRNA fluctuated throughout VPA treatment, in contrast with untreated patients in which the level was fairly stable, indicating that at least some response to the VPA treatment was obtained.

In this study we included only SMA patients with types II and III, avoiding the inclusion of the most severe form of the disease (type I). However, unfortunately we did not measure the *SMN2 *copy number in our patients that would be very interesting to divide the patients in more homogeneous groups and for a better comparison of our results with future studies.

According to Swoboda et al [26] the best potential gain from therapeutic intervention, if any, should be more probable in pre-clinic phases when the children have a large number of still healthy motor neurons, thereby allowing for more benefits. Swoboda et al (2009) [20] showed a significant improvement that was almost restricted to participants less than 5 years of age following VPA treatment in 42 patients with SMA. In our study, patients younger than six years of age had a better mean score on HFMS than the group older than six years of age, however, the comparison of the HFMS score of the first evaluation with the subsequent ones in the both groups (younger and older than 6 years of age) did not show significant differences. Moreover, in our study we observed a significant improvement of the HFMS scores during the VPA treatment in the group of type II children but not in the type III group, differently of the study of Weihl et al [18] that showed a more efficacious of VPA treatment in adult SMA patients than in younger ones with a more severe phenotype.

We routinely added L-carnitine to VPA therapy as there are numerous references to the potential benefit of this drug in preventing or even treating a possible hepatotoxicity of VPA due to its interference with mitochondrial beta-oxidation [27-30]. In addition VPA monotherapy depletes both muscle and serum carnitine levels [30]. Side effects were unremarkable in our study. Five patients complained of weight gain, and other patients displayed increased tremor of their fingers after six months of VPA intake, as observed by Tsai et al [19] in one 5-years-old child.

## Conclusion

Patients with type II and III SMA submitted to treatment with VPA during the period of one year had no significant side effects, and an increased of the motor abilities could be observed in children with type II SMA. Treatment of SMA patients with VPA may be a potential alternative to alleviate the progression of the disease.

## Competing interests

The authors declare that they have no competing interests.

## Authors' contributions

IAD participated in the design of the study, literature review, manuscript draft, and performed the functional assessment of the patients. PGP and MBDR participated in the clinical evaluation of the patients. EZ reviewed the manuscript and took part in the drafting of the final document. UCR coordinated the study and reviewed the manuscript. All authors read and approved the final manuscript.

## Pre-publication history

The pre-publication history for this paper can be accessed here:

http://www.biomedcentral.com/1471-2377/11/36/prepub

## References

[B1] Mostacciuolo ML Danieli GA Trevisan C Muller E Angelini C Epidemiology of spinal muscular atrophies in a sample of the Italian population Neuroepidemiology 1992 11 34 38 10.1159/000110905 1608493

[B2] Thieme A Mitulla B Friedemann S Spiegler AW Epidemiological data on Werdnig-Hoffmann disease in Germany (West-Thuringen) Hum Genet 1993 91 295 297 10.1007/BF00218278 8478016

[B3] Munsat TL Workshop report: International SMA Collaboration Neuromuscul Disord 1991 1 81 83 10.1016/0960-8966(91)90052-T

[B4] Zerres K Schoneborn S Forrest E Lusakowska A Borkowska J Petrusewicz I A collaborative study on the natural history of childhood and juvenile onset proximal spinal muscular atrophy (type II and III SMA): 569 patients J Neurol Sci 1997 146 67 72 10.1016/S0022-510X(96)00284-5 9077498

[B5] Lefebvre S Burglen L Reboullet S Clermont O Burlet P Viollet L Benichou B Cruaud C Millasseau P Zeviani M Identification and characterization of a spinal muscular atrophy-determining gene Cell 1995 80 155 165 10.1016/0092-8674(95)90460-3 7813012

[B6] Parsons DW McAndrew PE Iannaccone ST Mendell JR Burghes AH Prior TW Intragenic telSMN mutations: frequency, distribution, evidence of a founder effect, and modification of the spinal muscular atrophy phenotype by cenSMN copy number Am J Hum Genet 1998 63 1712 1723 10.1086/302160 9837824 PMC1377643

[B7] Velasco E Valero C Valero A Moreno F Hernandez-Chico C Molecular analysis of the SMN and NAIP genes in Spanish spinal muscular atrophy (SMA) families and correlation between number of copies of cBCD541 and SMA phenotype Hum Mol Genet 1996 5 257 263 10.1093/hmg/5.2.257 8824882

[B8] McAndrew PE Parsons DW Simard LR Rochette C Ray PN Mendell JR Prior TW Burghes AH Identification of proximal spinal muscular atrophy carriers and patients by analysis of SMNT and SMNC gene copy number Am J Hum Genet 1997 60 1411 1422 10.1086/515465 9199562 PMC1716150

[B9] Prior TW Swoboda KJ Scott HD Hejmanowski AQ Homozygous SMN1 deletions in unaffected family members and modification of the phenotype by SMN2 Am J Med Genet A 2004 130 307 310 10.1002/ajmg.a.30251 PMC4349519 15378550

[B10] Wirth B Brichta L Schrank B Lochmüller H Blick S Baasner A Heller R Mildly affected patients with spinal muscular atrophy are partially protected by an increased SMN2 copy number Hum Genet 2006 119 422 428 10.1007/s00439-006-0156-7 16508748

[B11] Swoboda KJ Prior TW Scott CB McNaught TP Wride MC Reyna SP Bromberg MB Natural history of denervation in SMA: relation to age, SMN2 copy number, and function Ann Neurol 2005 57 704 712 10.1002/ana.20473 15852397 PMC4334582

[B12] Kernochan LE Russo ML Woodling NS Huynh TN Avila AM Fischbeck KH Sumner CJ The role of histone acetylation in SMN gene expression Hum Mol Genet 2005 14 1171 1182 10.1093/hmg/ddi130 15772088

[B13] Brichta L Hofmann Y Hahnen E Siebzehnrubl FA Raschke H Blumcke I Eyupoglu IY Wirth B Valproic acid increases the SMN2 protein level: a well-known drug as a potential therapy for spinal muscular atrophy Hum Mol Genet 2003 12 2481 2489 10.1093/hmg/ddg256 12915451

[B14] Sumner C Huynh T Markowitz J Perhac JS Hill B Coovert DD Schussler K Chen X Jarecki J Burghes AH Taylor JP Fischbeck KH Valproic acid Increases SMN levels in spinal muscular atrophy patients cells Ann Neurol 2003 54 647 654 10.1002/ana.10743 14595654

[B15] Andreassi C Angelozzi C Tiziano FD Vitali T De Vincenzi E Boninsegna A Villanova M Bertini E Pini A Neri G Brahe C Phenylbutyrate increases SMN expression in vitro: relevance for treatment of spinal muscular atrophy Eur J Hum Genet 2004 12 59 65 10.1038/sj.ejhg.5201102 14560316

[B16] Tsai LK Tsai MS Ting CH Li H Multiple therapeutic effects of valproic acid in spinal muscular atrophy model mice J Mol Med 2008 86 1243 1254 10.1007/s00109-008-0388-1 18649067

[B17] Brichta L Holker I Haug K Klockgether T Wirth B In vivo activation of SMN in spinal muscular atrophy carriers and patients treated with valproate Ann Neurol 2006 59 970 975 10.1002/ana.20836 16607616

[B18] Weihl CC Connolly AM Pestronk A Valproate may improve strength and function in patients with type III/IV spinal muscle atrophy Neurology 2006 67 500 501 10.1212/01.wnl.0000231139.26253.d0 16775228

[B19] Tsai LK Yang CC Hwu WL Li H Valproic acid treatment in six patients with spinal muscular atrophy Eur J Neurol 2007 14 e8 e9 10.1111/j.1468-1331.2007.01992.x 18028187

[B20] Swoboda KJ Scott CB Reyna SP Prior TW LaSalle B Sorenson SL Wood J Acsadi G Crawford TO Kissel JT Krosschell KJ D'Anjou G Bromberg MB Schroth MK Chan GM Elsheikh B Simard LR Phase II open label study of valproic acid in spinal muscular atrophy PLoS One 2009 4 e5268 10.1371/journal.pone.0005268 19440247 PMC2680034

[B21] Swoboda KJ Scott CB Crawford TO Simard LR Reyna SP Krosschell KJ Acsadi G Elsheik B Schroth MK D'Anjou G LaSalle B Prior TW Sorenson SL Maczulski JA Bromberg MB Chan GM Kissel JT Project Cure Spinal Muscular Atrophy Investigators Network SMA Carni-val trial part I: Double-blind, randomized, placebo-controlled trial of L-carnitine and valproid acid in spinal muscular atrophy PLos one 2010 5 e12140 10.1371/journal.pone.0012140 20808854 PMC2924376

[B22] Zanoteli E Maximino JR Reed UC Chadi G Spinal muscular atrophy: from animal model to clinical trial Funct Neurol 2010 25 73 79 20923604

[B23] Main M Kairon H Mercuri E Muntoni F The Hammersmith Functional Motor Scale for Children with spinal muscular atrophy: a scale to test ability and monitor progress in children with limited ambulation Eur J Paediatric Neurol 2003 7 155 159 10.1016/S1090-3798(03)00060-6 12865054

[B24] Scott OM Hyde SA Goddard C Dubowitz V Quantitation of muscle function in children: a prospective study in Duchenne muscular dystrophy Muscle Nerve 1982 5 291 301 10.1002/mus.880050405 7099196

[B25] Cid-Ruzafa J Damian-Moreno J Valoración de la discapacidad física: el índice de Barthel Rev Esp Salud Pública 1997 71 127 137 9546856

[B26] Swoboda K Kissel J Crawford T Bromberg MB Acsadi G Danjou G Krosschell KJ Reyna SP Schroth MK Scott CB Simard LR Perspectives on clinical trials in spinal muscular atrophy J Child Neurol 2007 22 957 966 10.1177/0883073807305665 17761650 PMC3260051

[B27] Silva MF Aires CC Luis PB Valproic acid metabolism and its effects on mitochondrial fatty acid oxidation A review J Inherit Metabol Dis 2008 31 205 216 10.1007/s10545-008-0841-x 18392741

[B28] Chan YC Tse ML Lau FL Two cases of valproic acid poisoning treated with L-carnitine Hum Exp Toxicol 2007 26 967 969 10.1177/0960327107087799 18375641

[B29] Lheureux PE Hantson P Carnitine in the treatment of valproic acid-induced toxicity Clin Toxicol (Phila) 2009 47 101 111 19280426 10.1080/15563650902752376

[B30] Anil M Helvaci M Ozbal E Kalenderer O Anil AB Dilek M *S*erum and muscle carnitine levels in epileptic children receiving sodium valproate J Child Neurol 2009 24 80 86 10.1177/0883073808321060 19168820

